# Risk Factors for Disease Recurrence in Women With Phyllodes Tumors of the Breast in Southern Colombia: A Nine-Year Cohort Study

**DOI:** 10.7759/cureus.7951

**Published:** 2020-05-04

**Authors:** Justo Olaya, Juan Sanjuan, Rina L Luna, Lucia Casanova

**Affiliations:** 1 Surgery - Mastology, Unidad Oncologica Surcolombiana, Neiva, COL; 2 Surgery - Mastology, Universidad Surcolombiana, Neiva, COL; 3 Surgery - Mastology, Hospital Universitario Hernando Moncaleano Perdomo, Neiva, COL; 4 Clinical Research, Utopiapp SAS, Cali, COL; 5 Clinical Research, Cirugia y Trauma (CYTRA) - Universidad Surcolombiana, Neiva, COL; 6 Surgery, Universidad Surcolombiana, Neiva, COL; 7 Surgery, Hospital Hernando Moncaleano Perdomo, Neiva, COL; 8 Pathology, Hospital Hernando Moncaleano Perdomo, Neiva, COL; 9 Cardiac Surgery, Clínica Medilaser, Neiva, COL

**Keywords:** breast disease, thoracic and breast oncology - areas of interest, phyllodes tumors, mastology

## Abstract

Introduction

Phyllodes tumors (PTs) are uncommon ﬁbroepithelial breast tumors that occur in middle-aged women, and they tend to vary in biologic behavior. Surgical management is the standard therapy for the condition, but factors associated with recurrence remain unclear. The aim of this study was to evaluate clinical and surgical characteristics related to PT recurrences.

Methods

This retrospective cohort study included patients in southern Colombia who were diagnosed with PT and managed at a level I teaching and referral hospital over a nine-year period. Factors associated with recurrence were determined by Cox regression analysis.

Results

This study included 61 patients; their median age was 46 years [interquartile range (IQR): 39-55 years]. Pathologically, 37 tumors (60.7%) were classified as low-grade. The median tumor size was 7 cm (IQR: 4-11.5 cm). Thirty-nine (63.9%) patients underwent quadrantectomy. Nine patients (14.8%) experienced tumor recurrence, with the median time to recurrence being one year (IQR: 0.5-2 years). Distant metastasis was observed in four patients (6.6%) at a median of nine months (IQR: 0.4-2.5 years). Univariate analyses showed that patients with high-grade tumors [hazard ratio (HR): 2.90, p = 0.148] and those who underwent mastectomy (HR: 2.90, p = 0.460) were at higher risk of recurrence.

Conclusion

PT recurrence may be associated with biological features, the extent of local excision, tumor size, and negative margins. However, multicenter data are needed to confirm these findings.

## Introduction

Phyllodes tumors (PTs) are rare ﬁbroepithelial neoplasms that compromise the mammary glands. Estimates have indicated that around 0.3-1% of all breast tumors in women are PTs [1]. PTs are more prevalent in women aged 40-50 years than in other age groups [1-3]. The World Health Organization (WHO) has classified these tumors according to their behavior and biological characteristics as benign, borderline, or malignant [4,5]. These tumors have also been classified according to their invading ability and recurrence, the latter being either local or distant [4,5]. Surgical resection is the gold standard therapy, with margins of ≥1 cm reducing the risk of tumor recurrence [6,7]. Also, adjuvant radiotherapy is recommended under the principles for treating soft-tissue sarcomas [6,7]. Features of PTs associated with disease recurrence remain unclear [8]. The present study was designed to assess the relationships between tumor recurrence and the clinical and surgical characteristics of patients diagnosed with PT who were treated in southern Colombia over a nine-year period.

## Materials and methods

Study features

Patients with PT who were diagnosed and treated at a level I academic and referral hospital in southern Colombia over a nine-year period from 2007 to 2015 were retrospectively analyzed. The medical records of these patients were reviewed. The study protocol was approved by the local Institutional Review Board.

Statistical analysis

The sociodemographic characteristics, clinical evaluation, histopathological proﬁle, management, disease recurrence, and mortality rate during follow-ups were analyzed. Categorical variables were summarized as absolute and relative frequencies. Continuous variables were reported as medians and interquartile ranges (IQRs). Recurrence was analyzed using the Kaplan-Meier method and compared using log-rank tests. The relationship between recurrence and clinical and surgical features was assessed by calculating hazard ratios (HRs) and 95% confidence intervals (CIs). A p-value of <0.05 was considred statistically significant.

## Results

Over the nine-year period, 61 patients of median age 46 years (IQR: 39-55 years) were diagnosed with PT. Tumors were located in the right mammary gland in 32 patients (52.5%) and the left mammary gland in 29 patients (47.5%). The median follow-up period was 61 months (IQR: 36-87 months). Histopathological analysis showed that 37 patients (60.7%) had benign tumors, 15 (24.6%) had borderline tumors, and nine (14.8%) had malignant tumors. The median tumor size was 7 cm (IQR: 4-11.5 cm; range: 2-50 cm). Surgical management included wide local resection in three (4.9%) patients, lumpectomy in 39 (63.9%), and simple mastectomy in 19 (31.2%). The three patients who underwent lumpectomy had positive surgical margins, with two of these patients having benign tumors and the third having a borderline tumor; therefore, their surgical margins were widened. Because of the persistence of compromised margins, one of these patients required a mastectomy.

**Figure 1:**
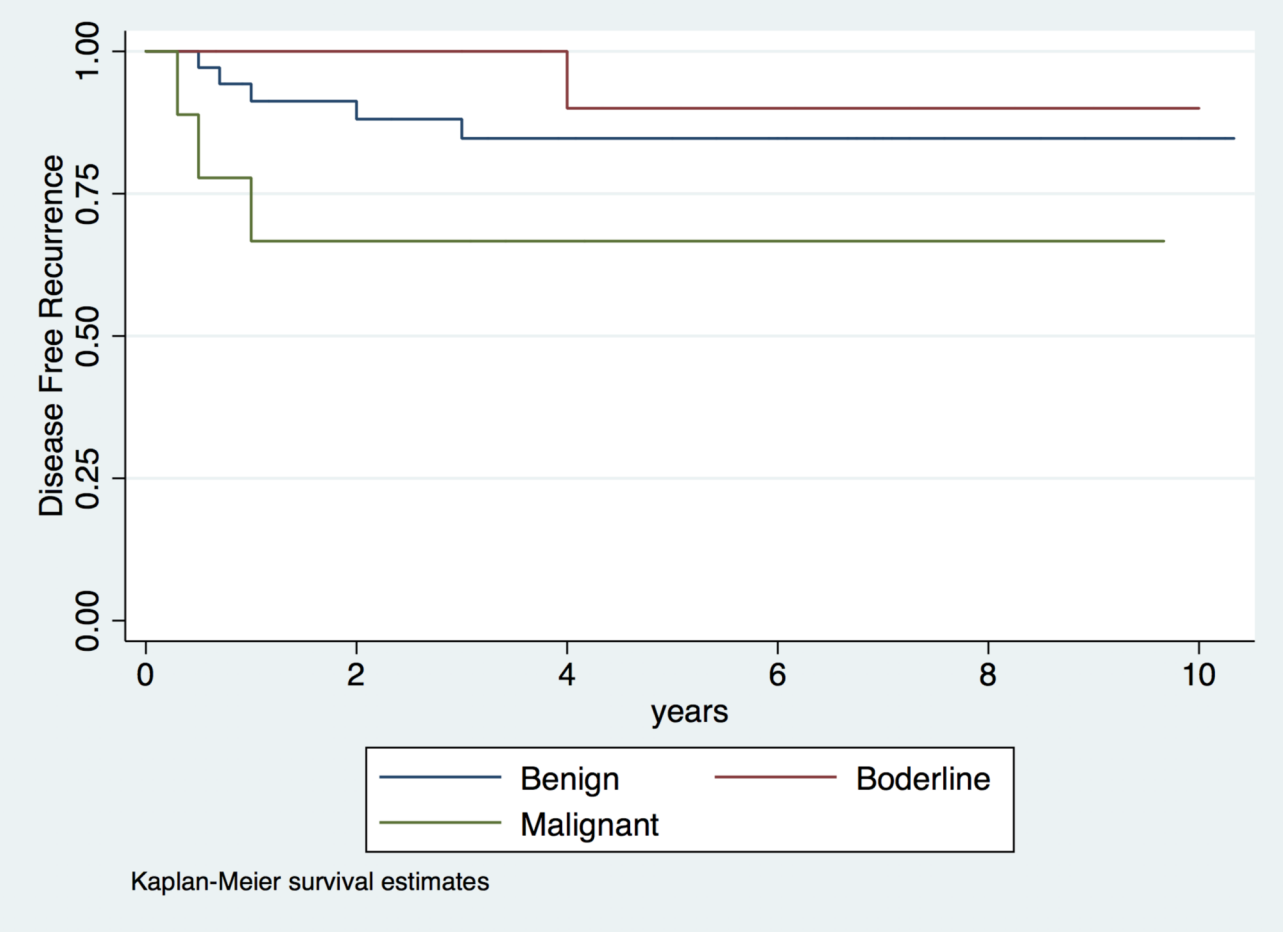
Risk of recurrence in patients with phyllodes tumors according to histologic characteristics

**Figure 2:**
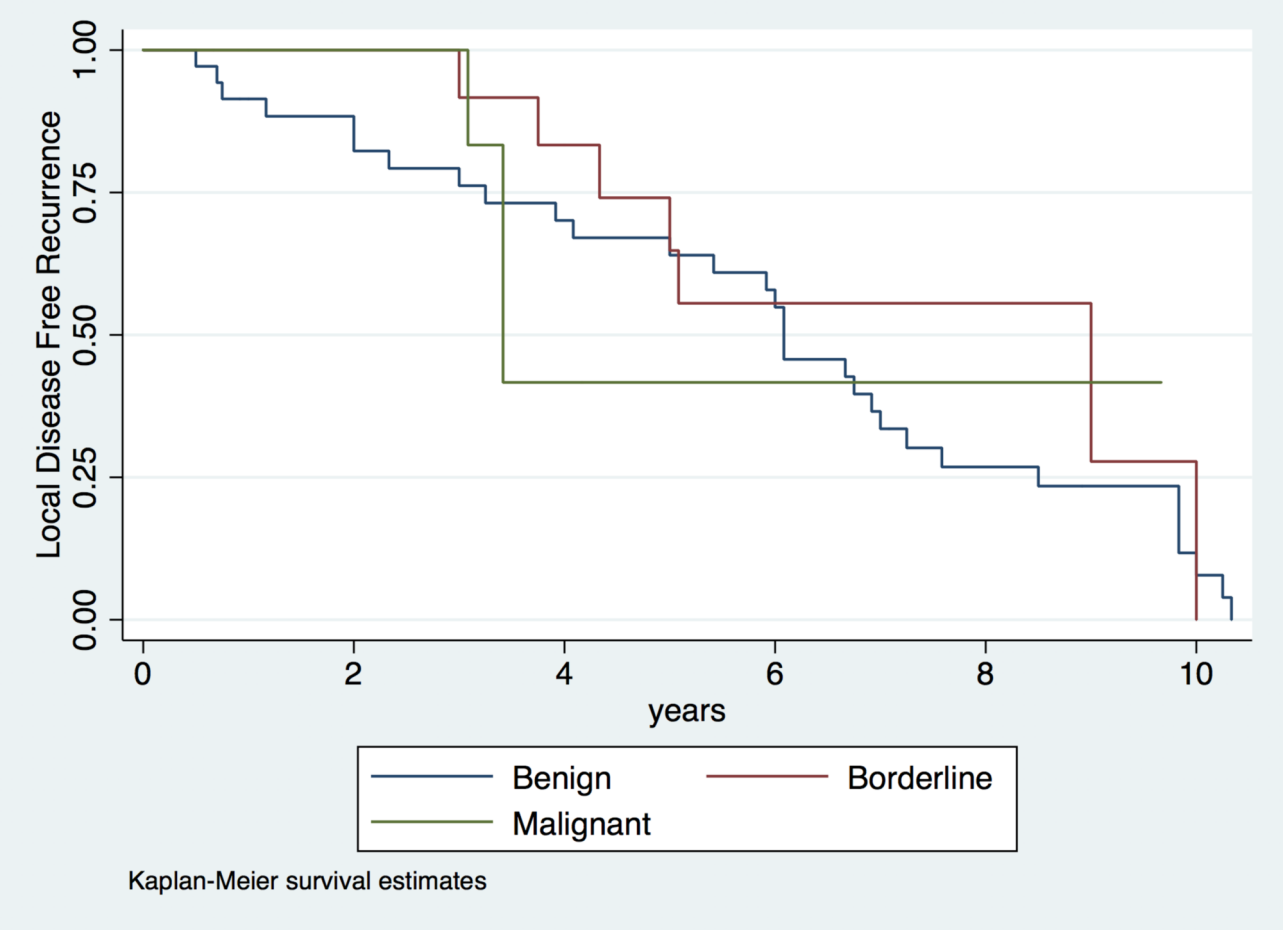
Risk of local recurrence in patients with phyllodes tumors according to histologic characteristics

**Figure 3:**
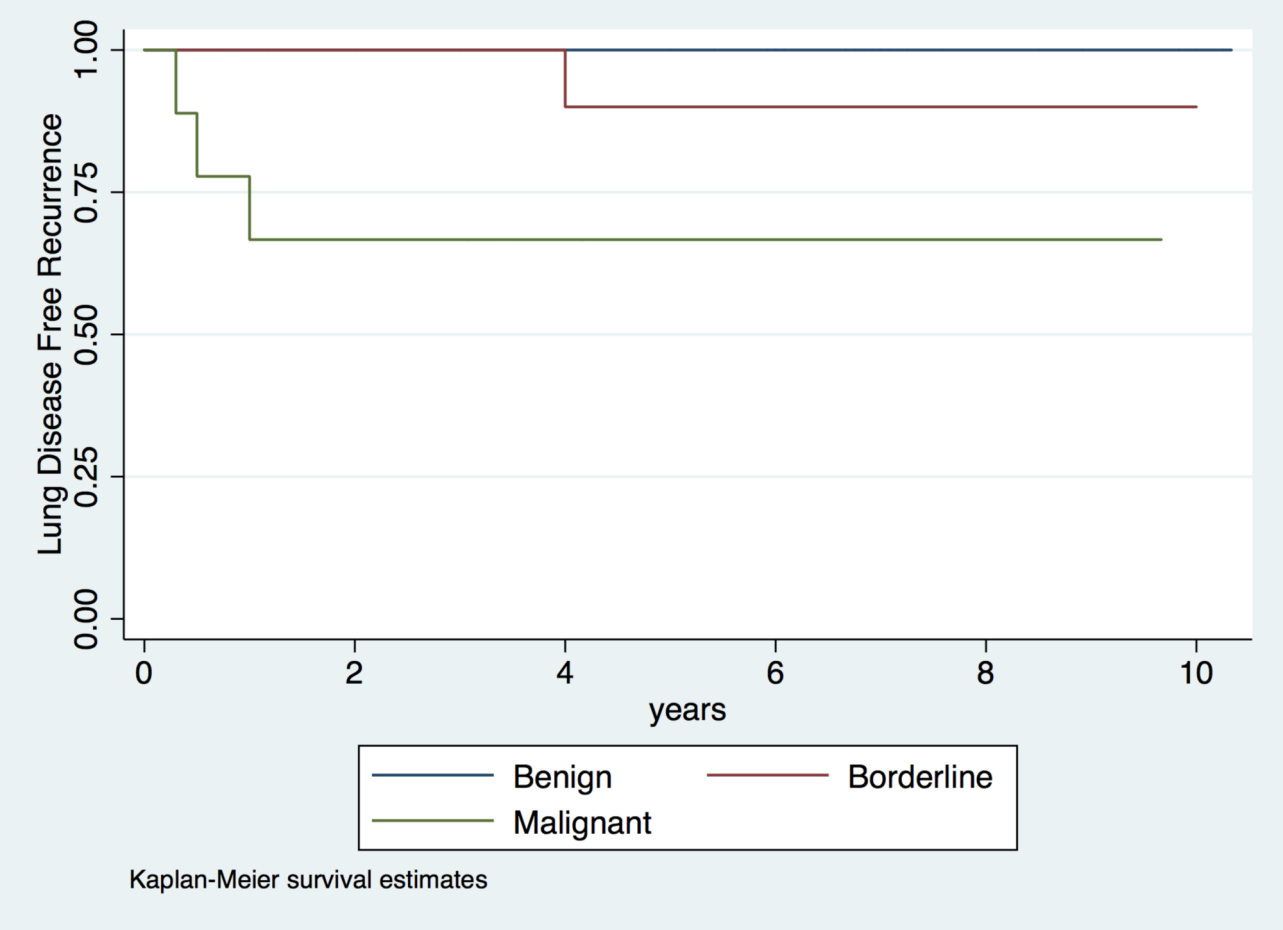
Risk of recurrent lung disease in patients with phyllodes tumors according to histologic characteristics

Disease recurrence was observed in nine patients (14.8%); the median time to recurrence was one year (IQR: 0.5-2 years; Figure 1). Five of these patients were diagnosed with benign tumors, one with a borderline tumor, and three with malignant tumors. The five patients diagnosed with benign tumors experienced local recurrence within a median time of approximately one year (IQR: 0.75-2 years; Figure 2). Initially, one patient underwent wide local resection, three underwent a lumpectomy, and one underwent a mastectomy due to a weak relationship between the mammary gland and the tumor (Table 1). The other four patients, one with a borderline tumor and three with malignant tumors, were diagnosed with distant recurrence and lung compromise at a median of eight months (IQR: 0.4-2.5 years; Table 2; Figure 3). All of these patients underwent a mastectomy.

**Table 1:** Relationship of histologic degree and size of phyllodes tumors with surgical management and disease recurrence

	Local resection (n = 42)	Mastectomy (n = 19)	Total
Tumor degree			
Benign, n (%)	4/32 (12.5%)	1/5 (20%)	5/37 (55.6%)
Borderline, n (%)	0/7	1/8 (12.5)	1/15 (11.1%)
Malignant, n (%)	0/3	3/6 (50.0)	3/9 (14.8%)
Tumor size			
<5 cm, n (%)	4/16 (20.0%)	0	4/20 (4.4%)
5–10 cm, n (%)	0/17	2/4 (50.0%)	2/21 (22.2%)
≥10 cm, n (%)	0/5	3/15 (20.0%)	3/20 (32.8%)

Univariate analysis results indicated that the risk of recurrence was higher in patients with malignant tumors (HR: 2.90; 95% CI: 0.69-12.25, p = 0.148) and in those who underwent radiotherapy (HR: 9.05; 95% CI: 2.42-33.8, p = 0.001; Table 2). The risk of recurrence tended to be higher in patients who underwent mastectomy (HR: 3.79; 95% CI: 1.02-14.14, p = 0.0.47) but was not associated with tumor size. Of the 61 patients, four (6.6%) died, all with distant recurrence.

**Table 2:** Hazard ratio for recurrent disease in patients with phyllodes tumors following surgery HR: hazard ratio; CI: confidence interval

	HR	95% CI	P-value
Mastectomy	3.79	1.02–14.14	0.047
Tumor degree			
Low	1		
Borderline	0.51	0.6–4.33	0.534
Malignant	2.90	0.69–12.25	0.148
Tumor size			
<5 cm	1		
5–10 cm	0.43	0.08–2.33	0.325
≥10 cm	0.90	0.20–4.03	0.889
Radiotherapy	9.05	2.42–33.8	0.001

## Discussion

The gold standard procedure for PTs consists of surgical management to control the wound and reduce recurrence [9]. Factors associated with disease recurrence may include biological behavior, tumor size, and surgical approach; however, the lack of randomized studies has prevented a definitive determination of factors associated with tumor recurrence [3,9]. The identification of such factors may guide treatment decisions and surgical approaches, resulting in better patient outcomes.

The present study analyzed patients with PTs who were treated at an academic and regional referral center in southern Colombia with an attention complexity level of III/IV. The medical histories of all patients were assessed to determine disease recurrence rates. Similar to previous reports, we found that the recurrence rate was 14.8%, and the mortality rate was 6.6% [2,3,5,8]. In line with other studies, we found that PTs were most prevalent in women in their fourth and fifth decades of life, although age was not a risk factor for disease recurrence [2,10,11]. Tumor sizes were larger than in previous reports, ranging up to 50 cm, although we found that tumor size was not a significant risk factor for recurrence, even after adjusting for histological features [2,11]. In contrast, a previous study found that median tumor size was significantly greater in patients with recurrence compared to those without recurrence (8.8 vs. 5.4 cm, p = <0.001) [10].

In contrast to the distribution of histopathological categories of PT behavior, we found that the proportion of patients with benign tumors was higher than in other international studies [2,10,12]. Histological features have been reported to be associated with the probability of disease recurrence [1,11]. In our study, some patients with PTs classified as benign experienced local recurrence, with one undergoing lumpectomy due to the persistence of compromised margins after undergoing surgery to widen margins. Distant recurrence was observed in some patients with borderline and malignant PTs, all of whom were treated with lumpectomy. This distribution was comparable to that in other studies [10-13]. Although stromal overgrowth has also been associated with the risk of disease recurrence, this factor was not included in our study [3,11].

A meta-analysis reported that local recurrence is significantly associated with surgical treatment, specifically with mammary-gland conserving surgery in patients with malignant PTs (odds ratio: 2.32; 95% CI: 1.01-5.30, p = 0.05) [11]. However, the surgical approach has been based on histological features, the tumor-to-mammary-gland ratio, and patient desire for breast preservation, indicating that recurrence risk is biased by an aggressive surgical approach relative to the differential risk of disease recurrence. Compromised resection margins have also been linked to local and distant recurrence [14-17]. However, these rates were lower in our study than in previous reports [2,3,8]. Indeed, only one patient in the present study had compromised resection margins, and this patient experienced a local recurrence.

Similar to previous studies, we found that the association between radiotherapy and disease recurrence was statistically significant (p = 0.001). Specific clinical and pathologic characteristics, including patient age, tumor size, type of surgery, and biological behavior may increase the likelihood of radiotherapy [2,18]. A recent meta-analysis suggested that the use of adjuvant therapy remains controversial, with the increased use of radiotherapy benefitting individual patients with PT, including younger women and patients with large-sized tumors, malignant histological behavior, and wide resection margins [18].

Our findings suggest that patients with malignant PTs are at higher risk of local and distant recurrence than patients with benign and borderline PTs. A nomogram illustrating the prognostic value and uncertainty of histological classification may provide a guide to the treatment of patients with PTs [8]. Stromal overgrowth should also be analyzed. The lack of inclusion of this feature in the current study is one of its main limitations. Another limitation was associated with the restricted analysis of subcategories due to the small sample size.

## Conclusions

To contribute to the understanding of PTs, we assessed clinical and surgical characteristics related to PT recurrences. Despite systematic reviews, some features related to disease recurrence remain unclear. One consistent finding has been that tumor size does not relate to recurrence, unlike histological features and surgical and radiotherapy approaches. Some factors might be related to the differential risk of disease recurrence.
